# Struggling to get started

**DOI:** 10.7554/eLife.59147

**Published:** 2020-05-27

**Authors:** Andy Tay

**Affiliations:** Department of Biomedical Engineering, University of SingaporeSingaporeSingapore

**Keywords:** COVID-19 and the Research Community, early-career researchers, grad school, postdoc, careers in science

## Abstract

As the world attempts to cope with the devastating impact of the COVID-19 pandemic, researchers about to start PhDs and postdocs face particular challenges.

The ongoing SARS-CoV-2 pandemic has had a massive impact on the research community. Research in a small number of fields related to the pandemic – notably epidemiology, public health, infectious disease, virology and respiratory medicine – has intensified. However, most universities and research institutes have been closed, forcing many researchers to stop ongoing experiments and, if possible, work from home. There has also been a knock-on effect on hiring and funding decisions: staff have been furloughed and many universities have announced hiring freezes (though many have also given extensions to tenure-track staff). But what impact has the pandemic had on scientists at the start of their careers, especially those hoping to start as graduate students later this year, and those looking for their first post-doctoral position? eLife spoke to three scientists in this position, and to two people working to help them.

## Waiting to get started

Fabian Schwerdtfeger started as a PhD student at Radboud University Medical Center in the Netherlands just two weeks before the lockdown in March. Since then he has been working at home on a literature review and learning to use image analysis software through online tutorials.

Schwerdtfeger is worried that the present lockdown, and any subsequent lockdowns or delays caused by future outbreaks of COVID-19, will limit the amount of experimental work he can do before his PhD contract ends. “In the Netherlands, one can only graduate with a PhD if one has a certain number of publications,” he says. “This is a problem because we could not do any experiments for the past few months and it may be some time before things become normal again. I hope to see the contracts of incoming and new PhD students being extended to make up for the lost time. Alternatively, schools should lower expectations concerning publications.”

At present Schwerdtfeger, who is from Karlsruhe in Germany, is not worried about not being able to see his family, but this could become a concern if the lockdown continues for more than a month. Schwerdtfeger is also grateful that his research group has been organizing virtual coffee hours as a way to maintain mental wellness during the pandemic. “You can read literature while you are at home, but reading eight hours a day for ten weeks is not possible. And it is worse when you know that you should actually be generating data, so the mental stress is not to be underestimated.”

## Worried about travel

Magdalene Ho hopes to graduate from Imperial College London in June and she has been accepted to start a PhD in bioengineering at Imperial later this year. She is currently in Singapore, where her family lives, and is taking her final-year exams virtually.

Ho’s main worry is that she might not be able to start her PhD in October if travel restrictions are still in place. She is also concerned that Singapore and the UK may have different definitions on what it means to be 'safe' to travel and work: “If the UK decides to reopen universities, but Singapore deems that it is unsafe to go to UK, will I still be able to return to Singapore during my PhD?”

Ho and others who are due to start their PhDs in bioengineering at the same time have been told by Imperial that they should be able to begin their PhDs in October and that they will be able to apply for extensions if their research is significantly hindered by the pandemic. “The bioengineering department has always prioritized student welfare so I am confident that there will be appropriate measures in place,” says Ho. And if she cannot travel to the UK in October, she plans to work on a literature review in Singapore and use Skype to keep in touch with her colleagues at Imperial.

## Uncertainties about funding

Max Cui is currently working as a research assistant at the University of Texas at San Antonio and has been accepted to start a PhD at the University of California, Berkeley later this year. However, she has only been offered a one-year fellowship, so one of her top priorities will be to secure funding for the rest of her PhD. “I am worried that if my research is delayed, I will not be as competitive to apply for funding next year,” says Cui. And with probable budget cuts, Cui is also concerned that her potential advisor might find it difficult to get grants to support her, and that other forms of income – from, for example, helping to teach undergraduate classes – might not be available either.

To complicate matters further, the lab that Cui hoped to work in was being renovated when the lockdown started, and there is little chance that the renovation work will be completed by the time her PhD is due to start. “I want to work on a project that most interests me, but I am worried about long delays. It’s a dilemma!”

## Waiting for answers

Universities are also struggling to answer questions from would-be PhD students and postdocs. “I have received many more questions than usual from anxious students regarding what fall quarter will look like and how visa processing will go,” says Allison Sherrill, a graduate student advisor at the University of Washington in Seattle. “My answers are that I do not know at this time, but that I will inform them as soon as I have reliable information.” Sherrill also acknowledges the challenges faced by those deciding where to do their PhD or postdoc, but adds that current graduate students and faculty have been generous with their time in arranging online meetings and interviews.

Barbara Natalizio, chair of the US National Postdoctoral Association (NPA), acknowledges that some institutions have asked principal investigators to be as accommodating as possible. However, she adds that others are “discouraging offers to new postdocs so as to not overcommit in a time of budget uncertainty”. The NPA also recognises the challenges faced by postdocs who need visas to work in the US: “The NPA hopes that the US Citizens and Immigration Services and related agencies will exercise discretion and flexibility to international postdocs as they navigate this unprecedented situation.”

